# The main physical components of body image from the perspectives of Iranian adolescents: a qualitative study

**DOI:** 10.1186/s12889-020-10096-7

**Published:** 2021-01-07

**Authors:** Sara Jalali-Farahani, Parisa Amiri, Fariba Zarani, Fereidoun Azizi

**Affiliations:** 1 Research Center for Social Determinants of Health, Research Institute for Endocrine Sciences, Shahid Beheshti University of Medical Sciences, Tehran, Iran; 2 Department of Clinical and Health Psychology, Faculty of Psychology and Educational Sciences, Shahid Beheshti University, Tehran, Iran; 3 Endocrine Research Center, Research Institute for Endocrine Sciences, Shahid Beheshti University of Medical Sciences, Tehran, Iran

**Keywords:** Body image, Body perception, Adolescents, Iran, Qualitative study

## Abstract

**Background:**

Although different aspects of body image have been extensively studied in Western societies, there is little evidence regarding the main physical components that contribute to perceptions of body image from the perspective of adolescents, especially in Eastern-Mediterranean regions. This study aims to investigate main physical components of body image from the perspectives of Iranian boys and girls**.**

**Methods:**

This qualitative study has been conducted using a grounded theory approach. The focus of the current study was on identifying the main physical characteristics of body image as perceived by participants. A total of 13 semi-structured focus group discussions were conducted with 84 adolescents (42 girls and 42 boys), aged 15–18 years. All focus group discussions were audio recorded and transcribed verbatim. Data collection continued until saturation was reached.

**Results:**

The mean age and BMI of participants were 16.2±0.9 years and 24.3±8.0 kg/m^2^ respectively. Regarding main physical components of body image, three main themes emerged from the data: 1) perceived face/hair, which included three subthemes-facial features, facial shape/form and hair, 2) perceived body, which included three subthemes-body size, body shape and bone structure and 3) perceived physical functioning which included three subthemes-physical ability, physical health and voice tone. Among mentioned themes, perceived body was the concept which was most frequently addressed by both adolescent boys and girls. Additionally, in terms of the other remaining two themes, when adolescents were talking about their bodies, girls more frequently mentioned their faces and its features, while boys tended to talk more about their physical functioning.

**Conclusion:**

This qualitative study provide further insight into the physical components of body image as perceived by Iranian boys and girls. Current findings indicated that besides those features that focused on body appearance and its aesthetic aspects, physical functioning was another main component of perceived body image by participants. These findings can contribute to the design and implementation of effective interventions aiming at improving body image and its related disorders in Iranian adolescents.

**Supplementary Information:**

The online version contains supplementary material available at 10.1186/s12889-020-10096-7.

## Background

Body image includes an individual’s perception, thoughts, feelings, and actions toward his/her body [1]. This topic has attracted the attention of many researchers in recent years and a considerable number of books, articles and dissertations have addressed this topic. Body image is strongly influenced by pubertal and identity development as well as physical and physiological changes that happen during adolescence [2]. Both positive and negative body image have been shown to influence health and health-related behaviors in adolescents. Existing evidence indicated that body appreciation, one of the main facets of positive body image is associated with lower disordered eating and alcohol and cigarette use and higher levels of physical activity and body-esteem [3–5]. On the other hand, body dissatisfaction and body concerns had a negative influence on several health-related behaviors including physical activity, dietary behaviors and tobacco and alcohol use [6–8]. Moreover, body dissatisfaction, which has been known as an important public health concern, found to be a risk factor for a broad range of physical and psycho-social health problems in adolescence [7, 9–11].

Different factors such as sex, body weight status, peers and family, social media and culture have been found to be associated with body image in adolescents [12–16]. Of the mentioned factors, culture can influence adolescents’ body image through various ways including its effects on the type and content of media that an individual is exposed to, common parenting styles in a society and ideal body characteristics that are valued in a community [17, 18]. Some evidence indicates that there is variation across different cultures regarding body size and body areas/parts that contribute to body dissatisfaction [19–22]. For example in one study, ideal body size differed across people of varying ethnicities in the U.S. with a larger body size being preferred by a higher percentage of Hispanic and Native American girls compared to their white counterparts [20]. In another cross-cultural study, although some body characteristics such as weight/shape; muscle; and upper, middle and lower body contributed similarly to body dissatisfaction in all male adolescents; Asian male adolescents found height, hair and face to contribute to their overall body dissatisfaction more compared to their Australian counterparts [21]. Although some components that contribute to body dissatisfaction may be universal; due to differences in cultural values in societies, the characteristics of an ideal body and body parts that get more attention may differ in various countries.

Though several studies conducted on different aspects of body image around the world, few of them focused on participants’ experiences and perceptions via qualitative approach. Existing qualitative studies on body image topic among adolescents are focused on exploring body ideals and their related socio-cultural determinants such as social media, parents and peers [23–27]; however, most of them conducted in Western cultures. As mentioned earlier, cultural values play an important role in shaping body image perceptions; hence, it is necessary to discover how related meanings are formed in non-Western cultures. In Iran, based on a recent review on body image topic, existing literature were mostly focused on adults (more than 70% of existing studies) and most of studies had cross sectional designs [28]. There were only two qualitative studies on body image topic in Iran which have been conducted on participants in their early adulthood [29, 30]. One of these studies explored influences of Instagram use on body image concerns of 24–34 years old female students [29]. The other qualitative study aimed to explore the ‘focus on the body and body change behaviors’ as a component of masculinity from the perspectives of 18–28 years male students, its findings demonstrated a new flexible type of masculinity as an ideal male body which was adopted from hegemonic masculinity and consumer masculinity [30].

Existing evidence on body image among Iranian adolescents, has mainly focused on the prevalence of body dissatisfaction and its association with other variables using a quantitative approach [31–35]. To the best of our knowledge, there is no study which explored different aspects of body image from perspective of Iranian adolescents. A qualitative approach can help researchers uncover unknown aspects of the phenomena under study that have yet to be understood using experiences of participants. There are different types of qualitative study designs. One of them is grounded theory approach which is different from traditional scientific model of research that use existing theoretical framework. As an inductive methods for conducting qualitative research, this approach allows researchers to explore the process of shaping a phenomena that is “grounded” in the lived experience of participants which may be different from those social context that studied previously. Considering existing gap on the literature, it is necessary to explore main physical components of body image from the perspectives of Iranian adolescents. Therefore, the current study employs a qualitative approach to uncover the perceptions of Iranian boys and girls regarding their bodies, with a focus on the main physical components that contribute to their body image perceptions.

## Methods

### Participants

This qualitative study conducted using a grounded theory approach. Study participants were 84 adolescents (42 girls and 42 boys) aged 15–18 years, who were recruited from high schools in Tehran (four all-boys schools and four all-girls schools). In an effort to obtain various experiences from different socioeconomic status, participants were selected from two different geographic areas in Tehran including north and south areas, representing areas of generally higher and lower socio-economic status, respectively. After compiling a list of Northern and Southern districts, using a simple random sampling method, one district was randomly selected in each geographical area. Then a list of all-boys and all-girls high schools located in each selected district was identified. Using simple random sampling, four high schools (two all-boys and two all-girls high schools) from each district were selected, for a total of eight high schools. In each selected high school, students were selected and invited to participate using a purposive sampling method. For selecting participants, school staff were asked to randomly select students with different physical features (e.g. height, weight and facial features) from various study majors and grades. Inclusion criteria required that participants be between 15 and 18 years of age; not have any chronic physical or mental diseases; agree to participate in group discussions; and provide written informed consents by both themselves and their parents. Of the students who were invited, the focus group discussions were scheduled for those who agreed to participate in the study and completed the consent forms. All of the students who were invited to participate in the study agreed to participate.

### Ethics

Ethics approval was obtained from the ethics committee of the Research Institute for Endocrine Sciences (RIES) of Shahid Beheshti University of Medical Sciences in Iran (IR.SBMU.ENDOCRINE.REC.1399.003). Informed consent was obtained from all participants and their parents prior to their involvement. Information sheets accompanied with parental consent forms were distributed among selected adolescents and they were asked to return completed forms to the researchers to begin participation. Permission to audiotape the focus groups was granted by participants. All participants were made aware that their identity and data would remain anonymous and confidential and that they could voluntarily withdraw from the study at any time without consequence.

### Data collection

Data was collected using semi-structured focus group discussions conducted by two female interviewers who had experience in conducting focus group discussions with adolescents. Focus group discussions are useful for the early exploration of a concept or topic because they harness the power of group dynamics to spark conversation and discovery of a broad topic. In focus group discussions, the researcher can facilitate conversation which can lead to feedback effect among participants. This group setting allows for the exchange of different perspectives between participants, obtaining fulsome findings. In addition, a focus group allows the researcher to gather more information in a shorter period of time. Considering the time constrains in a school setting, focus group discussions were selected to allow for the collection of diverse perspectives and enriched conversation in a shorter period of time with minimal disruption to students’ studies. Prior to the initiation of the discussions, the interviewers introduced themselves and informed participants of the aim of the study. They further explained that participants are expected to share their views and experiences regarding body image with a focus on their body characteristics. Data was collected between October 2018 and May 2019 through 13 same-gender focus group discussions with 6–8 participants in each focus group. The duration of focus group discussions ranged from 60 to 90 min. To ensure trustworthiness in the current study, participants were selected from two genders, various study majors and weight levels (underweight, normal weight, overweight/obese) as well as socio-economic statuses to enrich the exploration of the study objective. In addition, to allow for a safe space for participants to openly discuss sensitive matters, all sessions were conducted in a private room in the schools with no school staff present. Furthermore, to provide the chance of building trust and becoming familiar with the setting and context, a prolonged engagement of researchers with participants was applied to gain their trust and establish rapport. It means, the researchers spent sufficient time in the field to understand the culture and social settings which could affect adolescents’ perceptions of their body. Throughout the sessions, the researcher paraphrased and summarized the participants’ statements, and asked them to confirm their accuracy. After each focus group discussion, participants were asked to complete a form which includes socio-demographic information and self-reported weight and height. Each discussion was initiated by this question: “What do you think is the meaning of the term *body image*?”. After participants answered this question, a brief definition of the term “body image” was provided by the interviewer. Then, the discussion continued with open-ended questions as provided in the interview guide which has been developed for the current study (Additional file 1). Some example of the questions are as follows:

- When was the first time you noticed and judged your body? Which body parts caught your attention?
- If you want to evaluate your body, what score would you give yourself on a scale of 0 to 20?
- Why did you give yourself that score?
- In your opinion what are the characteristics of an ideal body in your age?
- If you could make a change in your appearance, what would that change be?

### Data analysis

Data collection and analysis were conducted simultaneously. All sessions were audio recorded and transcribed verbatim by the first author. In addition, notes were taken by a researcher about the nonverbal cues that participants exhibited. Data collection continued until the researchers believed saturation was reached, in which no new themes were observed. Data analysis was done manually using constant comparative analysis according to the Strauss and Corbin analysis method and open, axial and selective coding were applied to the data [36]. The constant comparative method was used by the researchers to develop concepts from the data by coding and analyzing at the same time. Using this method, potential themes and sub-themes emerged from the raw data. This study presents findings achieved through open and axial coding. Open coding was initially used by breaking up data into smaller parts and understanding the core idea of each part before assigning codes. Next, the emergent concepts and categories from the open coding process were linked together in categories and subcategories through axial coding [37]. For example, the codes “body waist and abdomen”, “body muscles” and “feminine curves” which emerged through open coding were labeled as “body shape”. Through the axial coding process, “body shape” and other related concepts such as “body size” and “bone structure” were considered sub-themes of a higher level theme labeled “perceived body”. All focus group transcripts were coded by the first author line by line. A review of the coded data and compatibility of assigned codes and concepts with quotations was conducted by three faculty members and a research assistant. If there was any disagreement in coding, the difference in perspective was resolved through discussion with the other researchers. Discussion between the research team members helped to explore various interpretations of the findings to reach agreements regarding final themes. Codes underwent respondent validation with a random subset of the participants and interpretation of findings were checked with participants from whom the data was obtained.

## Results

The mean age and BMI of participants were 16.2±0.9 years and 24.3±8.0 kg/m^2^, respectively. The majority of participants had married parents and about half of both mothers and fathers of participants had academic degrees. More than two thirds of participants’ mothers were housewives and the majority of participants’ fathers were employed. Residential, paternal and maternal characteristics of study participants are presented in Table 1.

**Table 1:** Descriptive statistics of study participants

	Boys (***n***=42)	Girls (***n***=42)	Total (***n***=84)
**Residential area n(%)**^**a**^			
- North of Tehran	20 (47.6)	28 (66.7)	48 (57.1)
- South of Tehran	22 (52.4)	14 (33.3)	36 (42.9)
**Marital status of parents n(%)**			
- Married	33 (84.6)	37 (90.2)	70 (87.5)
- Divorced/widowed	6 (15.4)	4 (9.8)	10 (12.5)
**Maternal level of education n(%)**			
- Primary	4 (10.3)	9 (21.4)	13 (16.0)
- Secondary	13 (33.3)	13 (31.0)	26 (32.1)
- Higher	22 (56.4)	20 (47.6)	42 (51.9)
**Maternal working status n(%)**			
- Housewife	25 (67.6)	30 (71.4)	55 (69.6)
- Employed	12 (32.4)	12 (28.6)	24 (30.4)
**Paternal level of education n(%)**			
- Primary	5 (13.5)	10 (24.4)	15 (19.2)
- Secondary	12 (32.4)	10 (24.4)	22 (28.2)
- Higher	20 (54.1)	21 (51.2)	41 (52.6)
**Paternal working status n(%)**			
- Employed	34 (91.9)	37 (90.3)	71 (91.0)
- Unemployed/retired	3 (8.1)	4 (9.7)	7 (9.0)

^a^North and south areas represents high and low socio-economic status, respectively

The body image components that emerged from the data were categorized into three main themes: 1) perceived face/hair, 2) perceived body and 3) perceived physical functioning. The first two themes are related to body appearance, while the latter refers to how body functions. Themes, subthemes, and codes are summarized in Table 2.

**Table 2:** Themes, subthemes, codes

Themes	Subthemes	Codes
**Perceived face/hair**	Facial features	- Size and shape of nose - Size, shape and color of eyes - Characteristics of mouth - Smoothness and color of skin
	Facial shape/form	- Jawline shape - Cheekbones shape - Natural face
	Hair	- Scalp Hair - Eyelash, eyebrow and beard
**Perceived body**	Body size	- Body weight - Body height
	Body shape	- Body waist and abdomen - Body muscles - Feminine curves
	Bone structure	- Neck length - Clavicle form - Knee angle - Shoulder width - Posture
**Perceived physical functioning**	Physical ability	**-** Physical readiness **-** Routine activities
	Physical health	**-** Lack of disability and illness - Abnormal symptoms
	Voice tone	None

### Theme 1: perceived face/hair

This theme refers to the main characteristics of adolescents’ face/hair which could affect their perception of and satisfaction with their body. These facial characteristics were categorized into three main subthemes including: 1) facial features, 2) facial shape/form and 3) hair. In the current study, girls more frequently focused on their facial appearance than did boys. Regarding facial features, most participants indicated that their nose, eyes, skin and mouth were the most important components of their face. Characteristics related to the nose and mouth were mostly referenced by girls, with a considerable number of girls commenting on the size and shape of their nose: *“I see some flaws in my face that I would like to change; for example, I don’t like my nose, it may need cosmetic surgery when I grow older”* (an adolescent girl). All comments related to characteristics of the mouth were stated by girls, which mainly focused on teeth “*If I have money, after doing vision correction surgery, I would spend the remaining on straightening my teeth*” (an adolescent girl)*,* and lips “*Beautiful lips must be plump”* (an adolescent girl)*.* On the other hand, characteristics related to skin and eyes were addressed by both girls and boys. In terms of eyes, participants commonly referred to size: *“I personally like a person who has pretty eyes, beautiful eyes like big eyes.”* (an adolescent boy) and color: *“I hate the dark color of my eyes”* (an adolescent girl)*.* Regarding skin characteristics, most participants commented on acne as an unpleasant factor that brought them dissatisfaction, for example: *“The first change that I would make in my body would be in my face, because I have acne on my face for almost 5-6 years, it’s always been a desire for me to use something like a sticker on my acne that would clear it up”* (an adolescent girl)*, and “But for a while now I am not satisfied with my face because of these pimples”* (an adolescent boy)*.*

Facial shape/form was another subtheme that was referenced frequently, such as jawline shape, cheekbone shape and natural face (i.e., not cosmetically enhanced). These characteristics were mentioned more frequently by girls than boys. Jawline shape was a characteristic that was mentioned by both girls and boys*: “For example, some individuals have angles on their faces, when you look at them this part of their face (the participant referred to the angles of her chin and ears) is angled.”* (an adolescent girl). Another comment made by a boy in reference to his jawline shape was: *“In terms of face form, I’m not very satisfied with myself at all, I like certain things that I don’t have, so I’m not satisfied. For example, an angled face.”* (an adolescent boy)*.* Cheekbone shape and natural face were two characteristics that emerged from comments made only by girls: “*An attractive girl has cheekbones”* (an adolescent girl)*,* and “*In my opinion, she [an Iranian actress] is very good, she is whatever she is, she doesn’t have any cosmetic surgery*” (an adolescent girl).

Another feature of the face that was pointed out by both adolescent boys and girls was hair. Adolescents mentioned characteristics related to the density and color of the hair on their head: *“I have hair loss and I cannot make a series of hairstyles because of my little hair, so I lose my body points because of it”* (an adolescent girl) and *“Another feature of an ideal body is blond hair”* (an adolescent boy)*.* In addition, facial hair including eyelashes, eyebrows and a beard were other characteristics that influenced adolescents’ perception of body image: *“Some individuals have very long lashes, some have very good eyebrows”* (an adolescent boy)*,* and *“He has a beautiful face, his beard is nice”* (an adolescent boy).

### Theme 2: perceived body

The second main theme that emerged from the data is perceived body which mainly focused on its shape and size. This concept was further categorized into three subthemes: 1) body size, 2) body shape and 3) bone structure. Body size, more specifically body weight and height, was the most frequently discussed subtheme by both boys and girls. In terms of weight, most adolescent boys and girls were dissatisfied with their perceived excess weight:*“ I hate my thighs, I hate my abdomen, I hate my sides, I hate all my body! I’m not obese in just certain areas, I am overall obese.”* (an adolescent girl) and *“I’m overweight, a good body has a weight and height that are proportionate, being muscular doesn’t matter.”* (an adolescent boy)*.* On the other hand, a few of them were dissatisfied with their thinness: *“I subtract my body score a bit because of being thin”* (an adolescent boy)*.* In terms of body height, boys and most girls indicated that a feature of an ideal body is tall height: *“If I wanted to give a score to my body, I would give 20 minus the score that people would give to height. If I was a step taller, I’d give it 20.”* (an adolescent boy). *“For example I’m short, my height is not tall enough, I’ve always liked to be tall”* (an adolescent girl)*.* However, a few girls were dissatisfied with their tall height and preferred to be shorter: *“I don’t like my height, I think I’m too tall”* (an adolescent girl)*.*

Another subtheme of perceived body was body shape which mainly relates to the waist and abdomen, body muscles and feminine curves. Waist and abdomen were referred to by both boys and girls with a higher frequency in girls: *“I am not very satisfied with myself for many years, for example, I sit down and say how big my abdomen is, it is really big! I mean for example when I wear tight clothes it seems that it is bigger than other parts”* (an adolescent girl)*.” The ideal body should have no abdomen, no flank!”* (an adolescent boy). Adolescents, primarily boys, referred to a muscular body as an ideal body: “*The body has to be in shape, because it’s not just about being lean. Sometimes thinness makes the body ugly, a thin body with muscles has a good shape, and an individual must go to gym and have toned muscle*.” (an adolescent girl). *“Actually, a good body should have a six-pack”* (an adolescent boy)*.* Feminine curves was exclusively mentioned by girls, in which bust and hips were areas that were emphasized: *“It is obvious, an attractive body should be like an hourglass!”* (an adolescent girl)*.*

With regards to the next subtheme of perceived body entitled as bone structure, comments made were related to different parts of skeletal structure. Some comments referring to neck length, clavicle form and knee angle were made only by girls: “*An ideal body should have a long neck*” (an adolescent girl), and “*An attractive body is a body with a distinct clavicle bone*” (an adolescent girl) and “*The knee angle and its shape is important. For example, it is important whether it is x-shaped, bowed or normal.*” (an adolescent girl). Shoulder width was a bone structure characteristic that was exclusively pointed out by boys “*I tried to create what I had in my mind, I was interested in body building and athletic body style and I tried to work out and broad shoulders was one of the things that mattered to me”.* (an adolescent boy). Finally, posture was another feature that was brought up by both adolescent girls and boys: *“It’s also really bad, like trying to wear something and you have a hump on your back! It’s so ugly!”* (an adolescent girl) and “*One point was my hunchback; for example, relatives said my mother, why does your son have a hunchback? And I was humiliated constantly*” (an adolescent boy).

### Theme 3: perceived physical functioning

In addition to face and body appearance, according to perspectives of current study participants, how the body functions influence adolescents’ perception regarding their bodies. The physical functioning theme had three main subthemes including 1) physical ability, 2) physical health and 3) voice. Physical ability referred to both physical readiness *“Whenever we had a race with other kids, I was out of breath because I’m overweight, it gave me a bit of trouble, I was always the last person”* (an adolescent boy*);* “*In my opinion, many of the effects of body shape are on body abilities rather than its appearance and beauty. I have understood this in my everyday life many times; for example, I went to the mountains with my family on holiday. Compared to my cousin who is very obese, I climbed up the mountain very quickly. Also my aunt who doesn’t exercise and get fit barely climbed up the mountain and she had a leg ache.”* (an adolescent girl) and ability for doing routine activities: “*Obesity is not really good in some situations, for example, if an earthquake happen, I am slower than thin people, I may fall under the rubble*” (an adolescent boy). In terms of physical ability, adolescent boys focused more on physical readiness compared to adolescent girls.

Another feature of physical functioning referred to adolescents’ assessment of their physical health which encompassed two main components: lack of disability and illness, and abnormal symptoms. From the perspective of adolescents, physical functioning was tied to lack of illness *“I don’t give a full score to my body, because of the problems I have, like headache or the diseases I have, including sinusitis”* (an adolescent girl) and disability “*I give myself a full score because I’m healthy, there are some people who don’t even have hands*” (an adolescent boy). In addition, abnormal symptoms was another feature of physical functioning which influenced adolescents’ perceived body image: “*I lose two points because I don’t have regular menstruation, for example, I menstruate every three months*” (an adolescent girl) which in some cases could also have negative effects on their social interactions “*I also lose 5 points for having sweaty palms, because for example when I get in touch with someone, I feel sweaty and it hurts a lot.*” (an adolescent boy).

Voice was a component of physical functioning that was mainly mentioned by boys: *“I am dissatisfied with my voice, for example, two years ago, my classmates made fun of my voice and said I sound like an addict”.* (an adolescent boy), and *“One of my friends has a six-pack and his voice is attractive, that’s why everyone pays attention to him. For example, once we went to the park, everyone gathered around him.”* (an adolescent boy).

## Discussion

The current study aimed to explore physical components of body image from the perspective of Iranian adolescent boys and girls. From this study, three main physical components of body image emerged from the data: perceived face/hair, perceived body and perceived physical functioning. Of these body image components, participants most frequently referred to perceived body (body shape and size) compared to the two other components. In addition, there was a gender difference in the findings with comments related to appearance (perceived face/hair and perceived body) being more prominent in girls’ statements, while comments related to physical functioning were more dominant in boys’ statements. In addition, comments made by several girls related to short height, a natural face, neck length, clavicle form and knee angle as well as comments made by boys related to voice tone were among the novel findings of the current study that are less addressed in the previous related research.

Based on the findings of the current study, perceived body was the most important component of body image and body features related to body size, shape and bone structure were frequently addressed in adolescents’ quotes. Of characteristics of perceived body, those features that reflected body size including body weight and height were prominent in adolescents’ statements implying that body size is a focal point in body perception of Iranian adolescents. In the current study, most of participants believed that a slim body, or a normal sized body, could be an ideal body. Furthermore, the majority of girls and all boy wished to be tall. These finding are consistent with findings from other parts of the world that have suggested a preference for thinness and tallness [38–43]. The preference for slim body by Iranian adolescents in the current study, is in accordance with thin idealization in Western culture. However, surprisingly, a minority of adolescent girls in the current study were dissatisfied with their tallness and preferred a shorter height.

Except for body size, participants pointed out to body features that reflect its shape. In this regard, a considerable number of girls referred to a curvaceous body as an ideal female body, while the majority of boys and few girls perceived a muscular body to be an ideal body. This finding is in agreement with findings of previous studies in which a curvaceous female body and a muscular male body were perceived as ideal body shapes by girls and boys respectively [44, 45]. Although evidence indicates both obese adolescent boys and girls are dissatisfied with their excessive weight, the mechanism of body dissatisfaction is more complex in boys. Existing evidence indicates a linear association between body dissatisfaction and BMI in adolescent girls implying thinness as an ideal body size in girls [46, 47]; whereas in boys, this association has been shown to be U-shaped where adolescent boys with both low and high BMI have shown high levels of body dissatisfaction [46, 47]. Body dissatisfaction among thin adolescent boys may reflect the cultural ideal for males to attain a traditionally masculine physique with muscles. This body ideal may explain why both underweight and obese boys are dissatisfied with their bodies; whereas thinness is more universally desired among girls.

Other than body size and shape, to a lesser extent, adolescents pointed out other features which were associated with their bone structure. Of these features, neck length, clavicle form and knee angle were mentioned by girls and shoulder width was mentioned by boys. In the current study, girls’ desires for having a long neck or distinct clavicles implies their preference for thinness; whereas, boys’ preference for having broad shoulders implies their desire for having a V-shaped figure. This finding is in agreement with existing literature indicating males’ preference for a V-shaped well-developed upper body characterized by having large biceps, chest, broad shoulders and narrow waist and hips [48, 49].

Posture was the only bone structure feature that was associated with body perception for both adolescent boys and girls. This finding implies that skeletal features contribute to adolescents’ perceptions of their bodies. These features are not confined to skeleton size and other features related to its shape including postural abnormalities are also important. In this regard, other spinal deformity namely idiopathic scoliosis found to associate with psychosocial problems including impaired social functioning, anxiety, body dissatisfaction, lack of self-confidence and feelings of anger, fear and shame in adolescents [50]. A study conducted in Iran demonstrated that kyphosis was associated with anxiety, depression and aggression in male high school students [51]. Postural kyphosis is a common problem among adolescents that can influence their physical and mental health. Reduction in levels of physical activity, spending many hours doing seated activities such as watching TV, slouching in front of a computer, playing on a tablet, sitting for many hours in school and carrying overloaded school bags are associated with the development of postural problems in school-age children [52, 53] Another factor that found to associate with postural problems in Iranian girls is breast development during puberty which has been associated with feelings of embarrassment [54] and can influence their posture. Therefore, modifying causes of postural problems is highly recommended to promote adolescents’ mental health.

Participants of this study believed that characteristics of their face and hair could influence their perception and satisfaction about their bodies as well. The focus on facial features was more prominent in girls’ statements compared to that of boys, implying that facial appearance is a more salient body-related concern in girls. In line with this finding, previous studies also indicated a gender difference pattern regarding body features that are emphasized by adolescent boys and girls [38].

Furthermore, current study found that some features such as big eyes, colored eyes, straight white teeth, plump lips, a small and upturned nose, smooth and bright skin, thick and long hair, and long eyelashes were perceived as desirable traits by participants, which is similar to previous studies [44, 55]. While most of the current study participants had dark-colored eyes and hair, having blue/green eyes, blond hair and an upturned nose were desired by them, which are more commonly idealized body features in Western societies [56]. Among facial features, the nose was most talked about among girls. This is not surprising, because based on previous reports, rhinoplasty is among the most commonly performed cosmetic surgeries in Iran and it is highly demanded by Iranians [57]. The high demand for rhinoplasty among Iranian girls may not only improve their perceived facial aesthetics, but may also be considered as a way to achieve a successful marriage and a financially powerful appearance [58]. To a lesser degree, characteristics related to facial shape, influenced participants’ perceptions regarding their bodies. These features included a sharper jawline according to both girls and boys, and high cheekbones and a natural face mentioned only by girls. Regarding the latter, a number of girls were opposed to cosmetic surgeries and believed that a natural face without cosmetic enhancement is more attractive than faces that have undergone cosmetic surgeries.

Irrespective of mentioned themes that mainly pointed out body appearance, a considerable number of participants alluded to physical functioning, specifically their physical ability, physical health and voice, as important aspects of their body image. In agreement with this finding, previous studies found that body image is not limited to body appearance and its aesthetic aspects, it is also impacted by perceived physical abilities, functions and health [19, 59, 60]. For example, Malaysian adolescents indicated their main reason for being concerned about their body shape is “to be healthy” (71%) rather than “to look good” (11.6%) [19]. Interestingly, in the study conducted by Abbot & Barber, the investigators pointed out that while functionality is less so considered in body image measurements, when it is integrated into related assessments in adolescents, its value transcends body appearance [60]. Whereas these findings indicate functionality is valued more than appearance by adolescents; findings of the current study suggested participants’ focus on their perceived body, a component of physical appearance, was higher than functionality. This may in part be attributed to increased access to the internet and photo-based social media sites such as Facebook, Instagram and Snapchat in recent years [61], providing the opportunity for adolescents to compare themselves and get feedback from peers about their appearance. Spending long hours on social media platforms may be accompanied by a stronger emphasis on physical appearance.

Physical function encompasses physical ability and physical health, both of which have been found to be associated with levels of physical activity and perception of physical fitness [62]. In addition, participating in sports can influence adolescents’ perception of their ideal body. For example, it was reported that the drive for muscularity-related behaviors were more common among adolescent boys who participated in weight-sensitive leisure sport compared to their counterparts who participated in less weight-sensitive leisure sport [63]. Therefore, levels and types of physical activity and physical fitness can influence adolescents’ vision of an ideal body, with more active adolescents and those with better physical fitness preferring a more muscular body. Finally, a more unique finding in the current study is the importance placed on voice by adolescent boys. Although this feature is mentioned less in previous studies, another study also found that adolescents referred to voice as a source of body dissatisfaction [42].

Among the novel findings of the current study were gender differences in perceived body image. For example, comments related to appearance (perceived face/hair and perceived body) were more prominent in girls’ statements, while comments related to physical functioning were more prominent in boys’ statements. This may be rooted in different body ideals and gender roles that are considered appropriate and desirable for each gender in society. Existing evidence indicates female body ideals are associated with thinness and signifiers of sexiness, including feminine curves; while male body ideals are associated with leanness and muscularity which implies emphasis on how a female body should look and how a male body should act [64]. The objectification theory explains this phenomenon, in that a high prevalence of objectifying images of females may promote self-objectification and self-monitoring in girls and women [65].

Finally, in present study, a negative tone was evident in participants’ statements and experiences which was accompanied by a higher focus on body dissatisfaction rather than body appreciation. This finding implies a high level of body dissatisfaction among participants which may be due to idealism and perfectionism which are common among this age group [66, 67].

The study findings provide beneficial information which can be applied by public health and health promotion specialists. This study expands our understanding of physical body features that attract the attention of adolescent boys and girls and provide helpful information for developing and revising existing screening tools used for the detection of body image concerns in adolescents. In addition, the findings demonstrate that both aesthetic and body physical functioning aspects were perceived important by both sexes to varying degrees; and these perceptions can influence different aspects of their health. Therefore, it is recommended to promote positive body image in adolescents, specifically overweight/obese ones. To improve adolescents’ physical functioning, it is highly recommended to incorporate lifestyle modification through health promotion programs. In addition, it is suggested to provide appropriate educational material and related training on topics of healthy eating habits and dietary intakes, good posture, disadvantages of sedentary behavior and benefits of physical activity to promote physical health and physical abilities. Similarly, teaching school staff and parents the importance of body image is recommended to foster a more positive environment for adolescents to learn from their role models and feel accepted.

This qualitative study is among the first to explore body image components perceived by Iranian boys and girls. A strength of this study is that male and female participants were selected from different socio-economic and weight status groups with the aim of maximizing variation in sampling. There are some limitations to this study. First, considering the nature of the focus group discussion, adolescents may have been influenced by their peers in the group, especially as participants were from the same school and some of them from the same grade and knew each other. In addition, participants were selected from an urban community; hence, current findings do not reflect perspectives of suburban and rural adolescents and further research in these settings is needed to broaden our understanding of this subset of Iranian adolescents. Finally, this study has been focused on the experiences of adolescents. To get a richer understanding of this topic, exploring the experiences and perspectives of parents, school staff, sociologists and related health specialist such as psychologists is recommended in future research.

## Conclusion

The current qualitative study provides insight into the physical features of body image which occupy the minds of Iranian adolescents and influence their body perceptions. Current findings revealed an overall negative tone in participants’ statements which reflect high levels of body dissatisfaction and highlight the necessity of promoting positive body image in future interventions. Based on the current findings, besides those features that focused on body appearance and its aesthetic aspects, physical functioning was another main physical component of perceived body image which was valued more than appearance by adolescent boys. This finding emphasizes the importance of encouraging and facilitating participation in physical activity programs as a strategy to improve body image, specifically in boys of this age group. Policy makers, health professionals and parents should pay special attention to adolescents with mentioned physical features such as excessive weight, acne and postural problems that could predispose them for developing body image problems and its negative health consequences. Finally, further exploration of the psychological aspects underlying perceived body image is highly recommended to provide a more comprehensive picture of perceived body image by this age group.

## Supplementary Information

**Additional file 1.** Interview guide. The semi-structured interview guide used for focus group discussions

## Data Availability

Data used in the current study would be available from corresponding author on reasonable request.

## Abbreviation

BMI: Body mass index
