# Supplementary material for: The main physical components of body image from the perspectives of Iranian adolescents: a qualitative study
Source: BMC Public Health. 2021 Jan 7;21:78. doi: 10.1186/s12889-020-10096-7 (PMC7791799; doi:10.1186/s12889-020-10096-7)
Supplement: Supplementary file 1 — Additional file 1. Interview guide. The semi-structured interview guide used for focus group discussions [file 12889_2020_10096_MOESM1_ESM.docx]

**Interview guide**

**-** To begin, can you describe yourself to me? What would you say if you wanted to describe yourself?

- When was the first time you noticed and judged your body? Which body parts caught your attention?

- If you want to evaluate your body, what score would you give yourself on a scale of 0 to 20?

- Why did you give yourself that score?

- Of all the features you have, why these features were important to you?

- In your opinion what are the characteristics of an ideal body in your age?

- If you could make a change in your appearance, what would that change be?

**-** What things or who influenced your body perception and your feelings about it?

- Family and relatives
- Friends
- Media
- Social networks

- Do the feelings you have about your body affect your overall satisfaction with yourself?

- How the feelings you have about your body affected your life?
